# A genetically informed prediction model for suicidal and aggressive behaviour in teens

**DOI:** 10.1038/s41398-022-02245-w

**Published:** 2022-11-21

**Authors:** Ashley E. Tate, Wonuola A. Akingbuwa, Robert Karlsson, Jouke-Jan Hottenga, René Pool, Magnus Boman, Henrik Larsson, Sebastian Lundström, Paul Lichtenstein, Christel M. Middeldorp, Meike Bartels, Ralf Kuja-Halkola

**Affiliations:** 1 Department of Medical Epidemiology and Biostatistics, Karolinska Institutet, Solna, Sweden; 2 Department of Biological Psychology, Vrije Universiteit Amsterdam, Amsterdam, the Netherlands; 3 Amsterdam Public Health Research Institute, Amsterdam University Medical Centres, Amsterdam, the Netherlands; 4 Division of Software and Computer Systems, School of Electrical Engineering and Computer Science KTH, Stockholm, Sweden; 5 Department of Learning, Informatics, Management and Ethics, Karolinska Institute, Solna, Sweden; 6 School of Medical Sciences, Örebro University, Örebro, Sweden; 7 Centre for Ethics, Law and Mental Health (CELAM), University of Gothenburg, Gothenburg, Sweden; 8 Gillberg Neuropsychiatry Centre, University of Gothenburg, Gothenburg, Sweden; 9 Child Health Research Centre, the University of Queensland, Brisbane, QLD Australia; 10 Child and Youth Mental Health Service, Children’s Health Queensland Hospital and Health Services, Brisbane, QLD Australia

**Keywords:** Human behaviour, Genomics

## Abstract

Suicidal and aggressive behaviours cause significant personal and societal burden. As risk factors associated with these behaviours frequently overlap, combined approaches in predicting the behaviours may be useful in identifying those at risk for either. The current study aimed to create a model that predicted if individuals will exhibit suicidal behaviour, aggressive behaviour, both, or neither in late adolescence. A sample of 5,974 twins from the Child and Adolescent Twin Study in Sweden (CATSS) was broken down into a training (80%), tune (10%) and test (10%) set. The Netherlands Twin Register (NTR; *N* = 2702) was used for external validation. Our longitudinal data featured genetic, environmental, and psychosocial predictors derived from parental and self-report data. A stacked ensemble model was created which contained a gradient boosted machine, random forest, elastic net, and neural network. Model performance was transferable between CATSS and NTR (macro area under the receiver operating characteristic curve (AUC) [95% CI] AUC_CATSS(test set)_ = 0.709 (0.671–0.747); AUC_NTR_ = 0.685 (0.656–0.715), suggesting model generalisability across Northern Europe. The notable exception is suicidal behaviours in the NTR, which was no better than chance. The 25 highest scoring variable importance scores for the gradient boosted machines and random forest models included self-reported psychiatric symptoms in mid-adolescence, sex, and polygenic scores for psychiatric traits. The model’s performance is comparable to current prediction models that use clinical interviews and is not yet suitable for clinical use. Moreover, genetic variables may have a role to play in predictive models of adolescent psychopathology.

## Introduction

Aggressive and suicidal behaviours cause significant disruptions on a personal and societal level [1–3]. Early identification of those at risk for these behaviours in the general population would likely improve prognosis by assisting with risk stratification as well as targeting clinical interventions, more efficiently than with clinical interviews for each child. However, many risk prediction models focus on clinical samples, i.e. those who have already sought treatment. By expanding this approach to the general population, prevention effectors could be improved. To this end, it is of interest to create a model that can determine who is at risk to exhibit suicidal behaviour, aggressive behaviour or both, versus who is not.

### Aggressive and suicidal behaviours

Aggressive behaviours are here defined as maladaptive behaviours that negatively impact others, such as hitting others or destroying property. Suicidal behaviours are considered to be a spectrum of behaviours that negatively impact the self, including: suicidal ideation, i.e. thoughts or planning of ending one’s life, suicide attempts, and death by suicide [4]. Although there is a debate on whether self-harm without suicidal intent is considered a suicidal behaviour, it is heavily implicated in suicidal behaviours and is a coping strategy borne from severe psychiatric symptoms which require clinical intervention. Thus, we included self-harm in our definition of suicidal behaviours due to its clinical relevance.

Suicidal and aggressive behaviours are often seen as separate constructs, however there is evidence for an intrinsic link [4–6]. Perhaps the most palpable example is in individuals with co-occurring symptoms of impulsivity and emotional dysregulation, such as with attention-deficit hyperactivity disorder (ADHD) or borderline personality disorder [7–9]. The occurrence of these symptoms can lead to impulsive-aggressive behaviour in a subset of individuals, blurring both the control and direction of their actions [10–13].

Many overlapping risk factors for these behaviours have been implicated across psychological symptoms and home environmental factors [14]. They have both been shown to be associated with internalising symptoms such as depression, substance abuse, family dysfunction and abuse during childhood, amidst a myriad of risk factors [7, 14–17]. Sex is a perhaps the most distinguishing risk factor as females are more likely to report self-harm, and suicidal ideation, while men have higher instances of death by suicide, aggressive behaviour and criminal acts [18–20]. There is evidence for genetic influence in the broad spectrum of suicidal behaviours and aggressive behaviours [21–23], though little is known regarding genetic overlap between them.

### Genetic overlap

Polygenic scores (PGS) represent an aggregate score of an individual’s genetic propensity for a trait based on effect sizes from genome-wide association studies (GWAS) and can be used as a measure of genetic risk for a trait, as well as for estimation of genetic overlap between traits [24]. With this approach genetic associations have been reported between suicidal behaviours and psychiatric traits like anxiety and depression [21], as well as between aggression and psychiatric traits including ADHD and autism spectrum disorder [22]. To the authors’ knowledge no such prediction models which examine suicidal and aggressive behaviours utilise these relatively novel methods. Thus, incorporating PGS for psychiatric traits may add information that improves prediction in suicidal behaviours and aggression.

### Predicting suicidal behaviours and aggressive behaviour

Many studies have looked into predicting suicide or aggressive behaviour. Although many models exist for prediction of suicidal behaviours in the clinical population, there are relatively fewer studies which use population-based samples [25, 26]. Whereas research in forensic psychology has worked to predict recidivism and violent criminal behaviour in general [27–29], an approach to prevent aggressive behaviours in adolescence, a developmentally sensitive period, is less common. Moreover, only a handful of studies using a clinical population have examined suicidal and aggressive behaviours together as an outcome [30, 31]. While aggressive behaviour tends to be childhood- and adolescent-limited, there is a subset of individuals for whom aggressive behaviour persists into adulthood. This trajectory is associated with poorer outcomes in adulthood [32]. Thus identifying those who remain aggressive at late adolescence or older are of clinical importance [33]. Additionally, given the significant overlap between the risk factors and co-occurrence of suicidal behaviours and aggression, creating a combined model that could be used in practice would reduce the need for separate questionnaires or assessments.

Our aim is to create a multi-class model that can predict who will report suicidal behaviours, aggressive behaviours, both, or neither in young adulthood within large scale, epidemiological samples. Using a combination of genetic, environmental, and psychosocial factors obtained from epidemiological cohorts would theoretically allow for a highly generalisable, comprehensive model that could improve understanding of risk factors for these behaviours, as well as further inform future models for clinical prediction and decision-making.

## Methods

### Participants

A total of 8676 participants from population-based twin cohorts, who completed self-report questionnaires about suicidal and aggressive behaviours between ages 17 and 21 were included in this study. Before the data cleaning procedures, the sample comprised 6,669 participants from the Child and Adolescent Twin Study in Sweden (CATSS) [34] and 2,764 participants from the Netherlands Twin Register (NTR) [35]. Further descriptions are provided in the Supplementary text.

### Measures

#### CATSS

Aggressive and suicidal behaviours were both measured using the Life History of Aggression Checklist [36] at age 18. Based on the average scores in a clinical sample, a score of 15 out of a possible 40 was used as a cut-off for determining aggressive individuals [36]. Suicidal behaviours were determined using two questions, “Deliberately attempted to injure yourself physically when you were angry or despondent” and “Deliberately attempted to kill yourself when you were angry or despondent”. Participants who endorsed either question were classified as having suicidal behaviours.

We included 19 predictors collected at age 9 or 12 and 15 which included psychiatric symptoms, parent and child relationship characteristics, as well as substance use [37–41]. A full list of variables and corresponding questionnaires used can be found in S1 and S2 Table.

#### NTR

Aggressive and suicidal behaviours were measured using the Young Adult Self Report and Adult Self Report of the Achenbach System of Empirically Based Assessment [42] at age 18. The aggression cut-off was derived using a T-score cut off of 64 from their respective aggressive behaviour subscales as suggested by the developers scoring system, which was equivalent to the 91.24% percentile of the NTR sample. Suicidal behaviour was measured using two related items from the internalising problems subscales: “I deliberately try to hurt or kill myself” and “I think about killing myself”. Participants who endorsed either question were classified as having suicidal behaviours.

The predictors, collected at age 12 and 16, overlapped with CATSS but were obtained from different questionnaires (S1 Table) [43].

### Polygenic scores

Polygenic scores (PGS) were constructed using summary data from recent GWASs. A complete list of the 17 traits and associated GWAS on which PGS were based can be found in S2 Table. Leave-one-out summary statistics excluding CATSS and/or NTR data samples were generated for any trait for which they were included in the discovery GWAS. Analyses were limited to individuals of European ancestry. From the psychiatric PGS scores a general psychopathology PGS variable was created to account for the underlying correlation between psychiatric disorders (S2 Table) [44, 45]. Genotyping and quality control were performed in both samples and are described in the Supplementary text.

PGS were derived using LDpred [46]. LDpred accounts for the linkage disequilibrium between single nucleotide polymorphisms (SNPs) to avoid inflation of effect sizes. For NTR the LD structure was determined on a subset of unrelated individuals and using a set of well imputed variants, while in CATSS, data from 1000 genomes phase 3 version 5 was used as an external reference sample [46, 47]. The weighted effect sizes were used as a basis for the polygenic scores. LDpred requires the specification of prior probabilities corresponding to the fraction of SNPs from the discovery samples considered causal with the trait, and we created scores at a range of priors (0.01, 0.05, 0.1, 0.2 0.3, 0.5, 1). In order to reduce the complexity inherent in having multiple PGS predictors and outcome variables, we performed principal component analysis (PCA) on all priors for each trait PGS, and included the first principal component (PCA-PGS) for each trait in our model according to Coombes and colleagues [48]. PCA analysis is an unsupervised machine learning technique which reduces the dimensionality of datasets while maintaining as much variability as possible; the resulting principal components (PCs) represent a certain amount of variation within the dataset. The first PC can be interpreted to represent the most variation within the data [49]. This method has been shown to prevent overfitting each PGS to each outcome and removes the need to select a single prior across all PGS.

### Data pre-processing

**Fig. 1:**
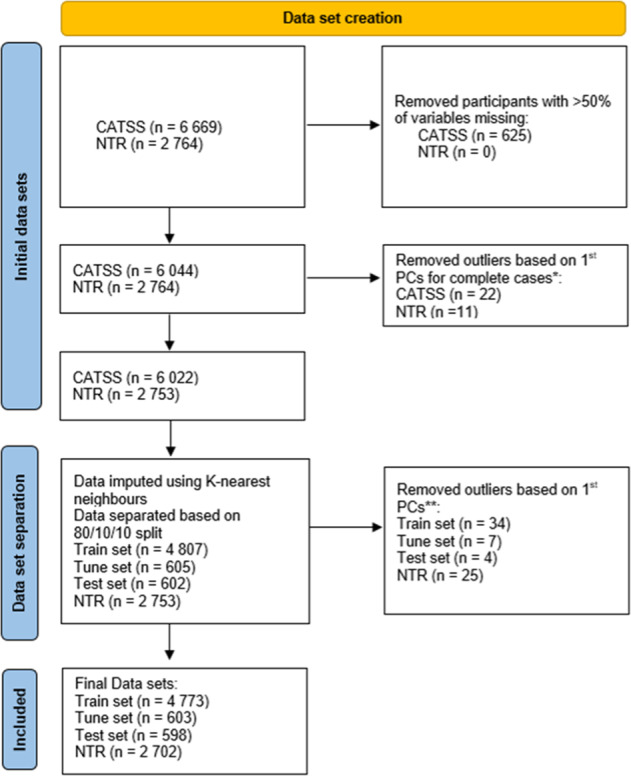
Flow chart of the data set creation. ^**^Principal component analysis (PCA) was completed separately for CATSS and NTR. Participants with a 1st principal component (PC) score outside −5.5–5.5 were used to determine outlier status. ^**^R package groupdata2 [65] removed 11 data points during the data separation process to preserve the proportion of the outcome between the sets. 3 separate PCA were completed to determine outliers: combined train and tune set, test set, and NTR. CATSS Child and Adolescent Twin Study of Sweden, NTR Netherlands Twin Register.

All analyses were performed in R. First, all non-binary predictor variables were scaled separately in CATSS and NTR to account for variations in measurement tools. Pearson correlation was completed on all included variables (S3 Table). Participants with more than half of variables missing were removed from the analysis (Fig. 1). This removal was for two reasons: (1) although a standard strategy is to maximise the number of data points this may not be the best approach when considering data quality; (2) K-nearest neighbours was used for imputation and requires a certain ratio of complete cases and removing those with more than half the data missing provided us with a suitable ratio.

Next, PCA was completed on the entire CATSS dataset to identify outliers which could hamper model performance [50]. Outliers were determined through the first principal component, severe outliers (scores outside the range of [−5.5, 5.5]) were removed. This process was repeated separately in the NTR dataset.

The CATSS data was then broken down into a training set, a tuning set, and a test set in an 80/10/10 split. As twin pairs are more similar to each other compared to other participants, we kept twins in pairs together in order to prevent overfitting that could occur from twin pairs being separated between the data subsets. Additionally, we stratified on the outcome to ensure that the outcome proportion was balanced across the data subsets. Descriptive statistics were checked to determine the consistency of the split. The NTR data was used without splitting.

Missing predictor data was imputed using K-nearest neighbours. A value of 6 was chosen for k, i.e. the number of data points within a cluster, was chosen by finding the square root of the number of columns in our dataset [51]. To avoid bias during this step, the test set and the external validation data were imputed separately. The outcome variable was not included as an informative imputation variable. We then completed a final check for outliers in the imputed datasets using the steps described above. This led to a total sample size of 8,676 participants (*N*_training_ = 4773; *N*_tuning_ = 603; *N*_test_ = 598; *N*_NTR_ = 2702) (Fig. 1).

### Statistical analysis

#### Main analysis

A PCA in the final data sets was completed to determine clustering of the four outcomes (suicidal behaviours, aggressive behaviours, both, or neither). R package H2O [52] was used for all supervised machine learning analyses. The performance of the models was determined by macro area under the receiver operating characteristic curve (AUC), derived from averaging the AUC for each class. The AUC for each class was determined using a one-versus-all approach that collapses each class into a binary outcome, e.g. having only suicidal behaviours vs all other outcomes combined [53]. Positive predictive values (PPV), negative predictive values (NPV), sensitivity, and specificity were determined based on Youden’s J statistic [54]. As the model merely gives probabilities of an individual belonging to the classes rather than giving a specific class prediction, a threshold must be selected at which a participant is classified into a specific class. Youden’s J is a way to determine this threshold by finding the maximum of the “sensitivity plus specificity minus 1”, associated with each of the possible thresholds; the values for J range from 0 to 1 [55].

First, a LASSO model without tuning was created to determine variable selection via coefficient weighting, variables which had a coefficient reduced to zero were considered to be unnecessary to the model and removed [56]. During the model creation process, each model was trained using the training set while the tune set was used to evaluate performance at each iteration. Neither the CATSS test set nor the external validation NTR dataset were used during this process. First, as the sample size for the classes varied extensively, we added weights to the training set to improve the performance of the model [57]. Weighting can be interpreted as the number of times participants in each class are resampled during the model learning process. The weight values *W* were determined by taking the sample size of the different classes over the sample size of the minority class (weights: *W*_suicidal behaviours_ = 6; *W*_aggressive behaviours_ = 10; *W*_both_ = 16; *W*_neither_ = 1). This means that during the learning process each model resampled individuals who had aggressive behaviours 10 times, while those in the majority class (neither suicidal nor aggressive behaviours) were used only once.

We created a stacked ensemble model, i.e. a model that combines input predictions from separate models, which included a gradient boosted machine, random forest, elastic net, and a neural network [58]. These models were selected based on their availability in H2O. This package was chosen based on its compatibility with multiclass ensemble models. The stacked ensemble model did not contain any parameters other than the number of folds for cross validation.

Each individual model was trained separately using cross-validation with 5 folds in the training set until the mean per class error converged (no improvements by 0.0001 for five rounds based on performance within the cross-validation folds). The model was then applied to the tuning set to determine if the model training process should continue. A combination of grid search and random search was used to tune the hyper-parameters of each model (S4–S7 Tables). Once the performance of each of the models did not improve after several rounds of parameter searching in the tune set, the models were combined into a stacked ensemble model, and the model was not further modified. Finally, we assessed the stacked ensemble model performance in the test set and in the NTR data.

Variable importance scores can be created from tree-based models and thus were obtained from the gradient boosted machines and random forest models that were included within the ensemble model. The overall variable importance rankings were determined using the average of scaled importance scores across the random forest and gradient boosted machine model. The variable importance in models built with H2O can be interpreted as the improvement in the squared error when the variable is split on a node, i.e. the decision points of the tree [52].

#### Sensitivity analysis

In order to determine the extent to which all of the PGS variables contributed to the model we completed the analysis with all genetic variables removed. The same tuning procedure in the main analysis was used to create the final ensemble model. We then performed a Venkatraman test using the R package pROC to determine whether the performance difference between both models (with and without genetic variables) was significant [59]. Additionally, in order to assess the discrepancy between the proportion of suicidal behaviours in the CATSS and NTR we performed a logistic regression using suicidal behaviours as an outcome with cohort and measurement year as predictors.

## Results

### Descriptive statistics

The data was fairly well-balanced between the training, tune, and test set (Table 1). The age of the sample at the measurement of the outcomes ranged from 17 to 21. As a sensitivity analysis we addressed the discrepancy between the percentage of those with suicidal behaviours and/or aggression in the sets, the CATSS data had a considerably higher number of individuals who reported suicidal behaviours (CATSS = 12.92%; NTR = 3.15%) or both aggression and suicidal behaviours (CATSS = 4.72%; NTR = 1.70%), while the NTR data had a higher number of individuals who were classified as aggressive (CATSS = 7.48%; NTR = 12.10%). In the logistic regression analysis, a statistically significant negative association with cohort (NTR coded as 1; *β* = −1.547; SE = 0.149; *P* < 2.00 × 10^−16^) and a non-statistically significant association with both birth year and the year of outcome measurement. This indicates that differences between cohorts, e.g. instruments, may explain the disparity in prevalence rates.

**Table 1:** Descriptive statistics for all datasets.

Description	N (% female)	Neither (%)	Suicidal behaviours (%)	Aggressive behaviours (%)	Both (%)	Average birth year (range)	Average year of outcome measurement (range)
Total data	8676 (59.43)	6717 (77.42)	857 (9.88)	774 (8,92)	328 (3.78)	1989 (1969–2000)	2012 (1990–2019)
NTR^a^	2702 (66.77)	2244 (83.05)	85 (3.15)	327 (12.10)	46 (1.70)	1986 (1969–1998)	2010 (1990–2014)
CATSS	5974 (56.11)	4473 (74.87)	772 (12.92)	447 (7.48)	282 (4.72)	1996 (1992–2000)	2014 (2010–2019)
Training set	4773 (55.75)	3587 (75.15)	617 (12.93)	348 (7.29)	221 (4.63)	1996 (1992–2000)	2014 (2010–2019)
Tune set	603 (61.36)	437 (72.47)	89 (14.76)	47 (7.79)	30 (4.98)	1996 (1992–2000)	2014 (2010–2019)
Test set	598 (53.68)	449 (75.08)	66 (11.04)	52 (8.70)	31 (5.18)	1996 (1992–2000)	2014 (2010–2019)

^a^The final proportion of individuals with aggression in the NTR does not correspond to the previously described T-score percentile because the T-score percentile was based on the total NTR sample while the final proportion is based on the sample after data cleaning.

The PCAs of the entire CATSS and NTR data sets did not show a clear distinction between individuals with and without the outcome (S1–S2 Figs.). No variables were reduced to 0 in the LASSO and thus none were removed from the model.

### Model performance

**Fig. 2:**
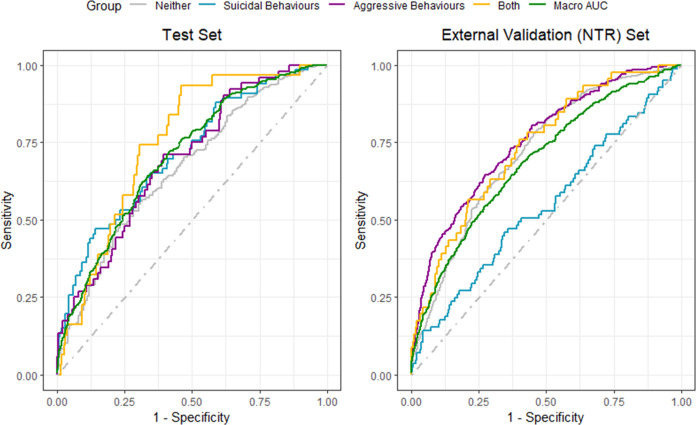
ROC curves for each data set. The macro AUC was derived from averaging the AUC for each class. The AUC for each class was derived using a one versus all approach, which collapses each class into a binary outcome, e.g. having only aggressive behaviour vs. all other outcomes combined. Test set (10,000 bootstrap; 95% CI): macro 0.709 (0.671–0.747); neither 0.667 (0.619–0.719); suicidal behaviours 0.713 (0.647–0.782); aggressive behaviours 0.696 (0.627–0.767); both 0.759 (0.696–0.829). NTR set: macro 0.685 (0.656–0.715); neither 0.715 (0.689–0.743); suicidal behaviours 0.543 (0.476–0.611); aggressive behaviours 0.751 (0.724–0.780); both 0.732 (0.662–0.807).

The performance of the four models included in the ensemble model were similar (S8 Table; final parameters S4-S7 Tables; the ensemble model contained no parameters): The ensemble model’s performance was relatively uniform between the datasets and classes, with the notable expectation of suicidal behaviours in the NTR, which was no better than chance (average of all classes, i.e. macro, AUC [10,000 bootstrap, 95% CI] AUC_CATSS(test set)_ = 0.709 [0.671–0.747]; AUC_NTR_ = 0.685 [0.656–0.715]; Fig. 2 and Table 2). The macro PPV was lower than the NPV (test set macro PPV = 0.350; test set macro NPV = 0.803; NTR set macro PPV = 0.303; NTR set macro NPV = 0.811; Table 2). Based on the Youden’s J statistic (test set = 0.306; NTR set = 0.278), the sensitivity (test set macro = 0.722; NTR set = 0.683) and specificity (test set macro = 0.584; NTR set = 0.594) of the models moderately varied. The confusion matrices for the training and test set can be found S9 Table.

**Table 2:** Model performance for the test and external validation sets.

	AUC (95% CI)^a^	Sensitivity	Specificity	Positive predictive value	Negative predictive value	Youden’s J statistic
*CATSS test set*						
Macro	0.709 (0.671–0.747)	0.722	0.584	0.350	0.803	0.306
Neither	0.667 (0.619–0.719)	0.486	0.765	0.862	0.330	0.251
Suicidal behaviours	0.713 (0.647–0.782)	0.470	0.859	0.292	0.929	0.329
Aggressive behaviours	0.696 (0.627–0.767)	0.712	0.604	0.146	0.957	0.316
Both	0.759 (0.696–0.829)	0.935	0.541	0.100	0.994	0.477
*External validation (NTR) set*						
Macro	0.685 (0.656–0.715)	0.683	0.593	0.303	0.811	0.276
Neither	0.715 (0.689–0.743)	0.784	0.541	0.893	0.339	0.326
Suicidal behaviours	0.543 (0.476–0.611)	0.459	0.654	0.041	0.974	0.113
Aggressive behaviours	0.751 (0.724–0.780)	0.645	0.727	0.246	0.937	0.372
Both	0.732 (0.662–0.807)	0.761	0.600	0.032	0.993	0.361

Youden’s J statistic was used to determine the optimal sensitivity and specificity. As models give probabilities of belonging to classes rather than specific class predictions, a threshold must be selected at which a participant is classified into a specific class. Youden’s J is a way to determine this threshold by finding the maximum result of the following equation with the values associated with each threshold: $sens + spec - 1$

The positive predictive value was determined based on the following equation: $\frac{{s{{{\mathrm{e}}}}ns \times prevalence}}{{\left( {sens \times prevalence} \right) + (\left( {1 - spec} \right) \times \left( {1 - prevalence} \right))}}$

The negative predictive value was determine based on the following equation: $\frac{{spec \times (1 - prevalence)}}{{\left( {(1 - sens) \times prevalence} \right) + (\left( {spec} \right) \times \left( {1 - prevalence} \right))}}$

The macro values were taken from the average values of each class, when collapsing each class down to a binary outcome.

^a^Area under the curve (10,000 bootstrap; 95% CI).

### Variable importance

**Fig. 3:**
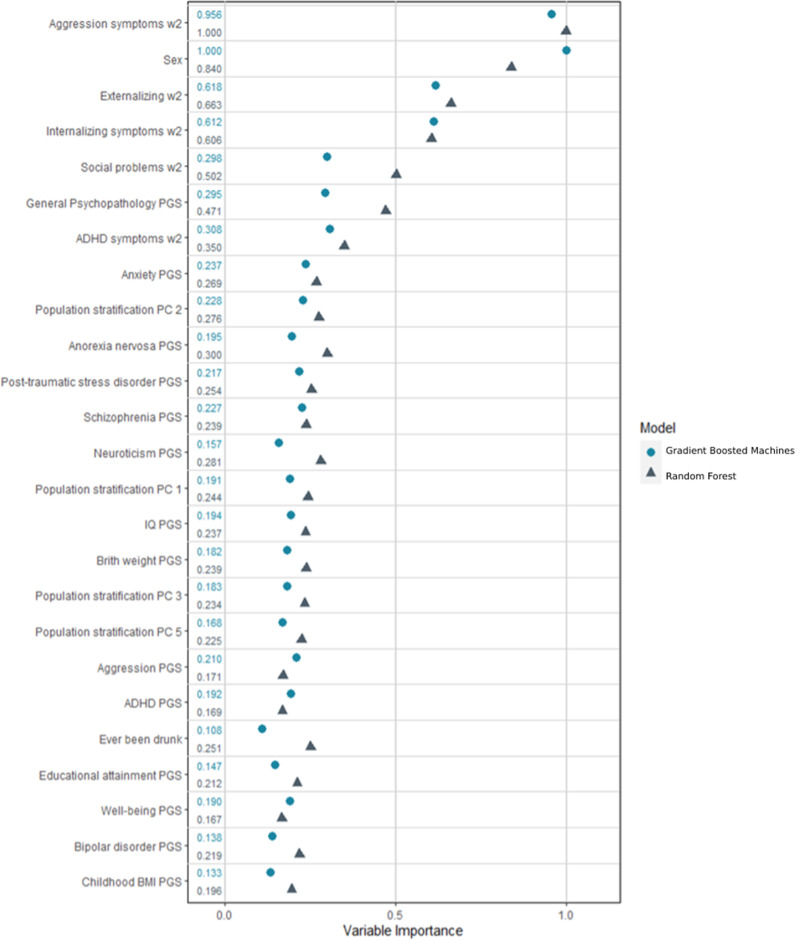
Scaled variable importance for the top 25 scores. Variable Importance in our model represents the reduction in mean squared error when the variable was split on a node; these values have been scaled for readability. Abbreviations: w2 = Measured at wave 2 (age 15/16); PGS = Polygenic score; PC = Principal component Gradient Boosted Machines Macro AUC tune set (10 000 bootstrap, 95% CIs): 0.653 (0.606–0.703); Random Forest Macro AUC tune set: 0.628 (0.580–0.678).

Variable importance rankings across gradient boosted machines and random forest were relatively consistent: self-reported aggression, sex, as well as externalising and internalising symptoms at age 15/16 were ranked as most informative (Fig. 3 and S10 Table). Several PGS were also ranked highly including psychiatric disorders, IQ, and childhood BMI/birth weight. Self-reported psychopathology measures consistently ranked higher than their corresponding parent-reported measures.

### Sensitivity analysis

The ensemble model which did not contain any genetic variables showed attenuated performance compared to the main model (macro AUC [10,000 bootstrap, 95% CI] AUC_CATSS(test set)_ = 0.677 [0.648–0.727]; AUC_NTR_ = 0.682 [0.653–0.710]) (S11 Table, final model parameters S12–S15 Tables). Testing the differences between both models showed that for most classes, there was no significant difference between the model with genetic variables and the model without (S16 Table).

The transparent reporting of a multivariable prediction model for individual prognosis or diagnosis (TRIPOD) Checklist [60] can be found in S17 Table.

## Discussion

We created a model to identify adolescents at a high risk of suicidal and/or aggressive behaviour using a wide range of predictors: questionnaires relating to home environment, behaviours, psychiatric symptoms, and genetic data for various traits. By training the model in the CATSS sample and validating it in the NTR sample, we tested the cross-cultural/external prediction of the model. Moreover, we examined the extent to which PGS variables contributed to the model through variable importance scores and by creating a model without genetic variables.

The AUC results for one-vs-all and macro, i.e. average of all one-vs-all scores, were similar between the CATSS test set (macro AUC = 0.709 [0.671–0.747]) and the NTR (macro AUC = 0.685 [0.656–0.715]). This indicates that the results of this model are generalisable across the national twin registers in the Netherlands and Sweden. Although the confidence intervals overlapped, the CATSS test set slightly outperformed the tune set (macro AUC = 0.697 [0.656–0.739]). This discrepancy came from a better performance of the suicidal behaviours and “both aggression and suicidal behaviours” classes. This be in part due to the slight variation in proportions between the classes, but counterintuitively, there were a lower proportion of individuals with suicidal behaviours in the test set (11.04% compared to 14.76%).

A cut-off of 0.8 for sensitivity and 0.5 for specificity has been previously proposed for use of prediction models in clinical settings [61]. The macro sensitivity (CATSS = 0.722; NTR = 0.683) did not meet this threshold, but the macro specificity did (CATSS = 0.584; NTR = 0.593). This criterion was met for “both aggression and suicidal behaviours” class in CATSS (sensitivity = 0.935; specificity = 0.541) but this did not hold true in the NTR, indicating that our model is not sufficient for clinical use yet. The PPV scores (macro PPV_CATSS(test set)_ = 0.350; macro PPV_NTR_ = 0.303) were lower than the NPV scores (macro NPV_CATSS(test set)_ = 0.803; macro NPV_NTR_ = 0.811). The macro PPV indicates that the model was correct around 30% of the time when it placed an individual into the four classes.

Overall, our study shows comparable to marginally improved prediction compared to previous studies investigating both suicidal behaviours and aggression outcomes in clinical settings. A previous study that developed a clinical risk assessment implemented by psychologists for self-harm and aggression had an average AUC of 0.68 and 0.66, respectively [30]. Another clinical study predicting self-harm and aggression in patients reported a PPV of 0.24 [31].

Similarly, the model’s performance was comparable to prediction models solely examining suicidal behaviours OR aggressive behaviours in clinical populations. The one-vs-all aggressive behaviours class had comparable performance to a study examining aggression in a prison sample [62]. The direct comparison for suicidal behaviours to other studies is more challenging due to varying definitions. Additionally, while the AUC for the over-vs-all suicidal behaviours class was satisfactory, the AUC for this class in the NTR was no better than chance. Therefore, our model under-performs in comparison to current models including those which make use of non-genetic biomarkers [61, 63]. That said, the macro results from this model moderately out performed or had comparable results to these studies.

By using a combination of data types we were able to investigate the performance of variables related to home environment, genetics, behaviours, and psychiatric symptoms. Our highest scoring variable was self-reported aggression at age 15/16, followed by sex, self-reported psychiatric symptoms at age 15/16, PGS of psychiatric traits including general psychopathology, anorexia nervosa, neuroticism, and anxiety disorders, as well as population stratification. The lowest ranked variables were related to family functioning and parent-reported symptoms. Our top variable importance scores fit within literature, as aggression is fairly stable in late adolescence [33]. Moreover, there are marked gender differences in the prevalence of the different outcomes [64, 65].

Out of the 25 highest variable importance scores 18 were PGS variables, with four PGS variables among the top ten. The highest performing PGS variables were for psychiatric related variables, but other traits such as birth weight, childhood body mass index, IQ, and educational attainment were also ranked highly. Additionally, removing all PGS variables from the model showed attenuated performance compared to the main model, however the difference in model performance was not statistically significant for most classes. Thus, while PGS of traits generally do not have clinical utility within psychiatry on their own, the results suggest that they can contribute to model performance when combined with other variables/risk factors [66]. Notably, AUC of the NTR outperformed the test set in the sensitivity analysis. This is likely a result of the model over relying on the variable with the highest importance, aggression at age 15/16, as the NTR had a higher proportion of individuals in the aggressive class and the one-vs-all aggression AUC was the top performing class in the NTR.

The primary strength of this study was the use of longitudinal data from both questionnaires and genetic data. Moreover, we were able to validate our model through an externally collected data source [67], indicating that our results are generalisable to Northern Europe and did not suffer from overfitting. However, our study comes with caveats. First, our model would likely be improved by a larger sample size and additional variables related to psychiatric symptoms, such as impulsivity and emotion-dysregulation. Second, our measure of suicidal behaviours were somewhat inconsistent across both cohorts, which likely affected the performance between the data sets.

## Conclusions

This study adds to the growing literature around genetically informed prediction models for mental health in adolescents. The results from the variable importance scores suggest that self-reported psychiatric symptoms in mid-adolescence, sex, and psychiatric PGS are key indicators for predicting later aggression and self-harm. Through the sensitivity analysis we found that removing genetic variables led to attenuated but not statistically significant differences in model performance for most of the classes. As of now prediction models are not ready for clinical use in psychiatric clinics and our model is no different [4]. Moreover, the current cost and processing time of genotyping means that clinical utility of genetically informed models may be limited, especially given our models comparable performance to models using non-genetic biomarkers [26].

Future studies could improve our results through including additional variables related to biomarkers, other psychiatric symptoms, and additional raters at varying time points. Moreover, investigating these predictors in a clinical sample could further improve the performance of the model. In sum, to our knowledge our study resulted in the first genetically informed population-model to predict suicidal behaviours, aggressive behaviours, their absence, and co-occurrence. In doing so, we created a model generalisable across Northern Europe with comparable performance to current models, (with the notable exception of the suicidal behaviours class). Finally, we show the non-inferiority of a model that used PGS variables in lieu of variables based on clinical interviews.

## Supplementary information

Supplemental file

S3 Table

S17 Table

## Data Availability

The code for the study is available upon request from the authors.
