# Supplementary material for: A genetically informed prediction model for suicidal and aggressive behaviour in teens
Source: Transl Psychiatry. 2022 Nov 21;12:488. doi: 10.1038/s41398-022-02245-w (PMC9678913; doi:10.1038/s41398-022-02245-w)
Supplement: Supplementary file 1 — Supplemental file [file 41398_2022_2245_MOESM1_ESM.docx]

**Cohort description and genotyping procedure**

**CATSS**

The Child and Adolescent Twin Study in Sweden (CATSS) is a longitudinal twin study targeting all twins born in Sweden since July 1, 1992. Parents are interviewed regarding their children’s mental health and behaviours in connection with their 9^th^ and 12^th^ birthdays, and at age 15 and 18, both twins and parents provide this information [1]. The CATSS has ethical approval from the Regional Ethical Review Board of Stockholm and all participants and parents provide consent.

Twins were genotyped using the Illumina Infinium PsychArray-24 BeadChip. Pre-imputation QC procedures have been previously described [2]. Genotype imputation was performed in Minimac3 using 1000 genomes data (Phase 3, Version 5) as the reference panel. Post-imputation QC included removing all SNPs with MAF <0.01, HWE p < 10^-6^, and call rate < 0.99. Additionally, individuals were excluded if the genotyping call rate was < 0.98.

**NTR**

The Netherlands Twin Register (NTR) has collected data on twins and their families, as well as families with new-born twins and triplets since 1987 [3]. The young NTR (YNTR) is made up of twins registered at birth by their parents. Information on twins under age 14 are provided by their parents and teachers, and from age 14 onwards, data collection is via self-report. Ethical approval was provided by the Central Ethics Committee on Research Involving Human Subjects of the VU University Medical Center, Amsterdam, an Institutional Review Board certified by the U.S. Office of Human Research Protections (IRB number IRB-2991 under Federal-wide Assurance-3703; IRB/institute codes 94/105, 96/205, 99/068, 2003/182, 2010/359) and participants provided informed consent.

Genotyping was done on multiple platforms including Perlegen-Affymetrix, Affymetrix 6.0, Affymetrix Axiom, Illumina Human Quad Bead 660, Illumina Omni 1M and Illumina GSA. For each genotype platform, samples were removed if DNA sex did not match the expected phenotype, if the PLINK heterozygosity F statistic was < -0.10 or > 0.10, or if the genotyping call rate was < 0.90. SNPs were removed if the MAF < 1^-6^, if the Hardy-Weinberg equilibrium p-value was < 1^-6^, and/or if the call rate was < 0.95. The genotype data for each platform was subsequently aligned with the 1000 Genomes reference panel using the HRC and 1000 Genomes checking tool, which tests and filters for SNPs with allele frequency differences larger than 0.20 as compared to the CEU population, palindromic SNPs and DNA strand issues. The data from all platforms was then merged into a single dataset, keeping all quality controlled SNPs of each platform. For each individual, one platform was chosen. Based on the ~10.8k SNPs that all platforms have in common, DNA Identity By Descent state was estimated for all individual pairs using the Plink and King programs. These estimates were then compared to the expected familial relations, and samples were removed if they did not fit. CEU population outliers, based on per platform 1000 Genomes PC projection with the Smartpca software, were removed from the data. Then, for each platform, the data was phased using Eagle and then imputed to 1000 Genomes and Topmed using Minimac following the Michigan imputation server protocols. Post imputation, the resulting separate platform Variant Call Format (VCF) files were merged with Bcftools into a single VCF file per chromosome for each reference, only for those SNPs present on all six platforms. For the polygenic scoring and parental re-phasing, the imputed data were converted to best guess data and were filtered to include only ACGT SNPs, SNPs with MAF > 0.01, HWE p > 10 ^-5^ and a genotype call rate > 0.98, and to exclude SNPs with more than 2 alleles. All mendelian errors were set to missing. The remaining SNPs represent the transmitted alleles dataset.

**Ancestry principal components**

Ancestry principal components in NTR and CATSS were generated by projecting study samples on PC axes derived in the 1000 genomes phase 3 version 5 reference sample [4] as follows:

1000G genotypes were restricted to the EUR, EAS, and AFR super-populations, except for the ACB and ASW populations within AFR (to avoid samples with likely recent admixture). Genotypes in each super-population group were filtered to only contain autosomal markers present in the HapMap3 reference data [5], with minor allele frequency (MAF) $\geq$ 0.01, Hardy-Weinberg equilibrium P-value $\geq$ 1.0*10-6, and missingness per sample and marker $<$ 0.02. Markers with alleles C/G and A/T were also excluded to avoid issues with strand alignment when merging datasets.

The markers present in all super-population groups after QC, and present in the quality-controlled imputed NTR data, were LD-pruned using plink (v1.90b6.24) [6] to have pairwise R2 $\leq$ 0.2 in 1000 kb windows, further excluding known regions of long-range LD [7].

A principal component analysis (PCA) was performed on the resulting marker set with plink (v1.90b6.24), using 1000G samples to define variant weights, and projecting the NTR samples (flags –within and –pca-cluster-names). Allele frequencies used in PCA calculations were derived in 1000G samples only.

CATSS samples were projected to the same PCA space as NTR samples using plink2 (v2.00a3LM AVX2 Intel [11 Oct 2021]) and hard-called imputed genotypes (flags –score no-mean-imputation variance-standardize), and each generated principal component variable was scaled by $1/\left( \sqrt{\lambda_{i}}/2 \right)$, where $\lambda_{i}$ is the corresponding eigenvalue, to match the scale of the NTR and 1000G samples.

The first five principal components were used as predictors in our model.

**S1 Table.** Included predictors

| **Description** | **Wave ^1^** | **CATSS sources** | **NTR sources** |
| --- | --- | --- | --- |
| Sex |  | Single item | Single item |
| Birth year |  | Single item | Single item |
| ADHD symptoms | 1 | ATAC | CBCL |
| Externalizing symptoms | 1 | ATAC | CBCL |
| Internalizing symptoms | 1 | ATAC | CBCL |
| Parents know child’s after school activities | 1 | SCM | FES |
| Social difficulties | 1 | ATAC | CBCL |
| ADHD symptoms | 2 | SDQ | YSR |
| Aggression | 2 | RPQ | YSR |
| Externalizing Symptoms | 2 | SDQ | YSR |
| Internalizing Symptoms | 2 | SDQ | YSR |
| Parent and child criticizes each other | 2 | PCRI | FES |
| Parent and child quarrel often | 2 | PCRI | FES |
| Social difficulties | 2 | SDQ | YSR |
| Has used marijuana | 2 | Single Item | Single Item |
| Has been drunk | 2 | Single Item | Single Item |

Note: ATAC, Autism-Tics, AD/HD and other comorbidities inventory [8]; CBCL, Child Behaviour Checklist [9]; FES, Family Environment Scale [10]; Parent Child Relationship Inventory [11]; RPQ, Reactive-Proactive Aggressive behaviours Questionnaire [12]; SCM, Statin Child Monitoring [13]; SDQ, Strength and Difficulties Questionnaire [14]; YSR, Youth Self-Report [15].

^1^ Ages at the waves for the cohorts were as follows:

CATSS: 9 & 12, 15; NTR: 12, 16

**S2 Table.** Included polygenic risk scores and genetic variables

| **Trait (reference)** | **Sample size** |
| --- | --- |
| ADHD [16] | 53,293 |
| Aggression [17] | 87,485 |
| Anxiety disorders [18] | 83,566 |
| Anorexia Nervosa [19] | 14,477 |
| Childhood-onset asthma [20] | 314,633 |
| Autism [21] | 46,350 |
| Bipolar disorder [22] | 51,710 |
| Birth weight [23] | 205,475 |
| Childhood BMI [24] | 39,620 |
| Educational attainment [25] | 746,714 |
| Head circumference [26] | 10,768 |
| IQ [27] | 269,867 |
| Major depressive disorder [28] | 332,580 |
| Neuroticism [29] | 390,278 |
| Post-traumatic stress disorder [30] | 174,659 |
| Schizophrenia [31] | 105,318 |
| Subjective Well-being [32] | 482,253 ^1^ |
| Population stratification principal components ^2^ | NA |
| General psychopathology score ^3^ | NA |

Note: ADHD, Attention-deficit hyperactive disorder; BMI, Body Mass Index; IQ, Intelligence Quotient

^1^ Rather than the entire well-being spectrum, summary statistics were recalculated to only include measures of life satisfaction and positive affect

^2^ The first five genetic principal components as predictors in our model were included to account for the population stratification in the data.

^3^ The general psychopathology score [33] was created by performing PCA analysis on the PCA-PGS scores related to mental health..

**S3 Table. Pearson correlation tables**

**S4 Table.** Parameters tried for random forest

| **Parameters** | **Description** | **Final** | **Range** |
| --- | --- | --- | --- |
| **Mtries** | Number of variables sampled at each node | 4 | 4 - 8 |
| **Max depth** | Maximum number of edges from the first to last node | 5 | 2-5 |
| **Number of trees** | Number of aggregated trees | 1000 | 200-1,400 |
| **Histogram type** | Method to break up continuous variables for the decision process | Round Robin | Round robin, Quantiles global, Automatic |
| **Sample rate** | Number of participants sampled in each tree | 1 | 0.7-1.00 |

Note: A combination of grid search and random search was used for the parameter search. The parameter search was stopped after all possible combinations were tried or if the mean per class error did not improve by at least 0.0001 for 5 rounds

AUC (1000 bootstrap, 95% CIs]) train set: Macro 0.841 (0.832-0.851) ; Neither 0.774 (0.759- 0.789); Suicidal behaviours 0.775 (0.756-0.794); Aggressive behaviours 0.884 (0.866-0.904); Both 0.931 (0.915-0.949)

AUC tune set: Macro 0.659 (0.619-0.700); Neither 0.628 (0.580-0.678); Suicidal behaviours 0.617 (0.559-0.677); Aggressive behaviours 0.722 (0.648-0.798); Both 0.668 (0.569-0.769)

**S5 Table.** Parameters for neural network

| **Parameters** | **Description** | **Final** | **Range** |
| --- | --- | --- | --- |
| **Hidden layers** | The number of nodes and layers | (20, 20) | (20, 20), (10,10,10), (50, 50), (30,30), (15,15,15), (5,5,5) |
| **Initial drop out ratio** | A form of regularization, i.e. minimizing loss, which randomly removes a ratio of layer outputs | 0.05 | 0 - 0.5 |
| **Rate** | Learning rate, the amount the weights are updated in response to the error rate | 0.01 | 0.01 – 0.02 |
| **Activation** | Input signal function for the node | Rectifier with dropout | Rectifier, Maxout, Rectifier with dropout, maxout with dropout, Tanh |
| **Epoch** | Number of iterations of the dataset | 50 | 1 - 100 |
| **L1** | Lasso regularization | 2.4^-5^ | 0 - 1^-6^ |
| **L2** | Ridge regularization | 9.7^-5^ | 0 - 1^-6^ |

Note: A combination of grid search and random search was used for the parameter search. The parameter search was stopped after all possible combinations were tried or if the mean per class error did not improve by at least 0.0001 for 5 rounds

H2o uses forward propagation

AUC (1000 bootstrap, 95% Cis) train set: Macro 0.731 (0.717-0.745); Neither 0.700 (0.683-0.717); Suicidal behaviours 0.693 (0.672-0.714); Aggressive behaviours 0.753 (0.727-0.779); Both 0.778 (0.747-0.810)

AUC tune set: Macro 0.665 (0.624-0.707); Neither 0.632 (0.584-0.681); Suicidal behaviours 0.656 (0.601-0.714); Aggressive behaviours 0.696 (0.619-0.781); Both 0.675 (0.579-0.776)**S6 Table.** Parameters for gradient boosted machines

| **Parameters** | **Description** | **Final** | **Range** |
| --- | --- | --- | --- |
| **Max depth** | Maximum number of edges from the first to last node | 3 | 2 - 12 |
| **Number of trees** | Number of aggregated trees | 500 | 250-10,000 |
| **Same rate** | Number of participants sampled in each tree | 1 | 0.20-1.00 |
| **Column sample rate** | Number of variables sampled aggregated with sample rate | 1 | 0.20-1.00 |
| **Column sample rate per tree** | Number of variables sampled at each tree, aggregated with column ample rate | 1 | 0.20-1.00 |
| **Column sample rate change per level** | Change of variable sampling per depth of tree | 1 | 0.9-1.10 |
| **Minimum rows** | Minimum number of participants in a node | 10 | 0-12 |
| **Nbins** | Number of bins for each histogram to build | 20 | 4 - 20 |
| **Learn rate annealing** | Rate to reduce to learn rate after each created tree | 1.0 | 0.5-1 |
| **Histogram type** | Method to break up continuous variables for the decision process | Automatic | Automatic, Quantiles global, Round robin |
| **Learn rate** | Learning rate, the amount the weights are updated in response to the error rate | 0.2 | 0.01-1.00 |

Note: A combination of grid search and random search was used for the parameter search. The parameter search was stopped after all possible combinations were tried or if the mean per class error did not improve by at least 0.0001 for 5 rounds.

AUC train set (1000 bootstrap, 95% Cis): Macro 0.956 (0.952-0.960); Neither 0.770 (0.756-0.785); Suicidal behaviours 0.777 (0.758-0.796); Aggressive behaviours 0.847 (0.827-0.868); Both 0.921 (0.904-0.939)

AUC tune set: Macro 0.670 (0.629-0.712); Neither 0.653 (0.606-0.703); Suicidal behaviours 0.643 (0.585-0.702); Aggressive behaviours 0.721 (0.640-0.809); Both 0.663 (0.567-0.766)

**S7 Table.** Parameters for elastic net

| **Parameters** | **Description** | **Final** | **Range** |
| --- | --- | --- | --- |
| **Alpha** | L1 and L2 regularization distribution | 0.9 | 0-0.90 |
| **Lambda** | Regularization strength | 0.008 | 0.001-0.01 |
| **Theta** | 1/x for the negative binomial family | 0.2 | 1^-10^ -0.1 |

Note: A combination of grid search and random search was used for the parameter search. The parameter search was stopped after all possible combinations were tried or if the mean per class error did not improve by at least 0.0001 for 5 rounds

AUC train set (1000 bootstrap, 95% Cis) : Macro 0.697 (0.682-0.711); Neither 0.680 (0.662-0.698); Suicidal behaviours 0.656 (0.633-0.678); Aggressive behaviours 0.712 (0.684-0.740); Both 0.740 (0.707-0.774)

AUC tune set: Macro 0.682 (0.642-0.724); Neither 0.654 (0.608-0.702); Suicidal behaviours 0.676 (0.620-0.734); Aggressive behaviours 0.731 (0.658-0.809); Both 0.668 (0.570-0.774)

**S1 Figure**. Principal component analysis for CATSS to determine distance between classes, principal component PC (variance explained)


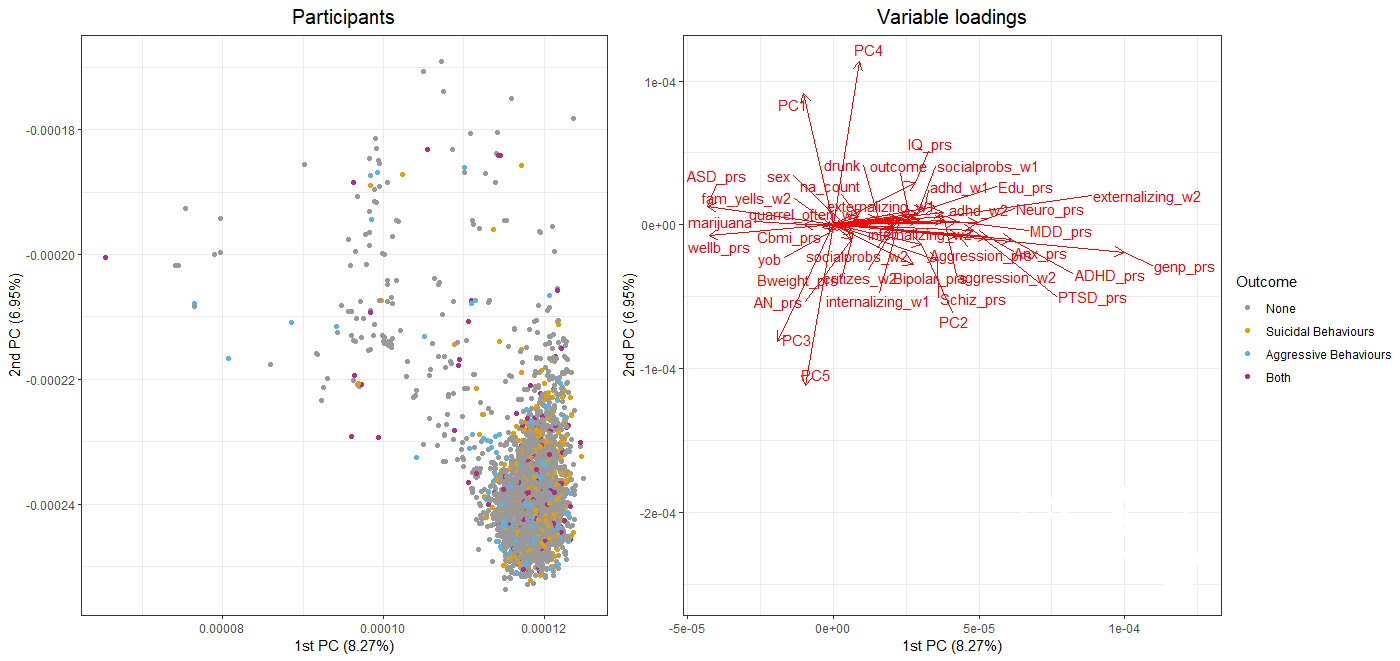


**S2 Figure**. Principal component analysis for NTR to determine distance between classes, principal component PC (variance explained)


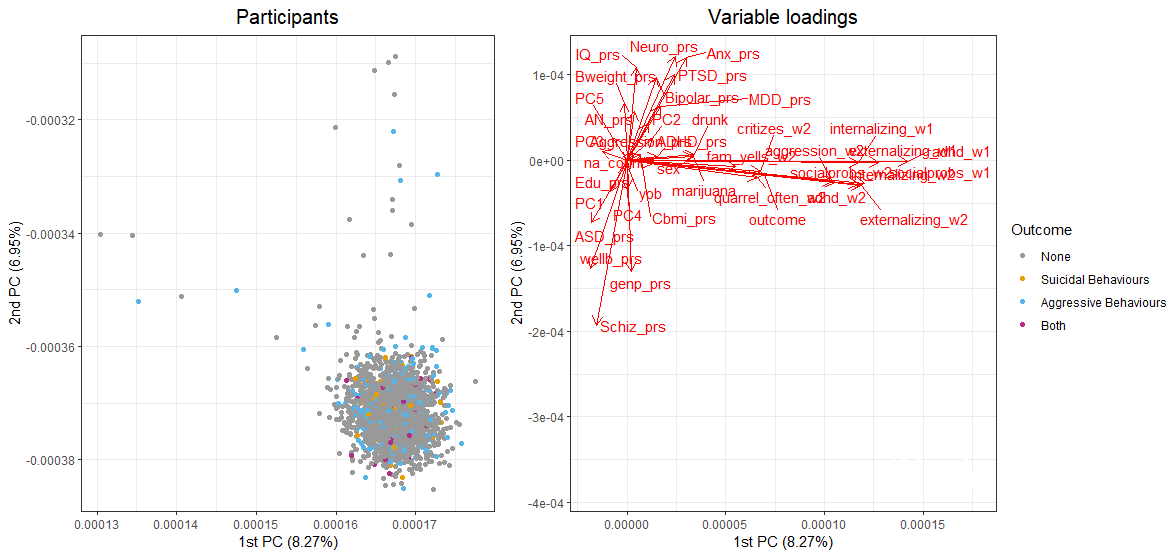


**S8 Table**. Area under the curves for the train and tune sets (10,000 bootstrap, 95% CIs)

|  | **Macro** | **Neither** | **Suicidal behaviors** | **Aggressive behaviours** | **Both** |
| --- | --- | --- | --- | --- | --- |
| **Tune Set** |  |  |  |  |  |
| Ensemble | 0.697 (0.56-0.739) | 0.668 (0.623- 0.715) | 0.689 (0.639-0.742) | 0.749 (0.677-0.827) | 0.681 (0.656-0.739) |
| Random Forest | 0.659 (0.619-0.700) | 0.628 (0.580-0.678) | 0.617 (0.559-0.677) | 0.723 (0.648-0.798) | 0.668 (0.569-0700) |
| Gradient Boosted Machines | 0.670 (0.629-0.712) | 0.653 (0.606-0.703) | 0.643 (0.585-0.702) | 0.721 (0.640-0.809) | 0.663 (0.567-0.766) |
| Elastic Net | 0.682 (0.642-0.724) | 0.654 (0.608-0.702) | 0.676 (0.620-0.734) | 0.731 (0.658-0.809) | 0.668 (0.570-0.774) |
| Neural Network | 0.665 (0.624-0.707) | 0.632 (0.584-0.681) | 0.656 (0.601-0.714) | 0.696 (0.619-0.781) | 0.675 (0.579-0.776) |
| **Train set** |  |  |  |  |  |
| Ensemble | 0.785 (0.773-0.798) | 0.737 (0.721-0.752) | 0.727 (0.706-0.748) | 0.835 (0.815-0.857) | 0.841 (0.817-0.867) |
| Random Forest | 0.841 (0.832- 0.851) | 0.774 (0.759 – 0.789) | 0.775 (0.756-0.794) | 0.884 (0.866-0.903) | 0.931 (0.915-0.949) |
| Gradient Boosted Machines | 0.829 (0.818-0.839) | 0.770 (0.756-0.785) | 0.777 (0.758-0.796) | 0.847 (0.827-0.868) | 0.921 (0.904-0.939) |
| Elastic Net | 0.697 (0.682-0.711) | 0.770 (0.756-0.785) | 0.656 (0.633-0.678) | 0.712 (0.684-0.740) | 0.740 (0.707-0.774) |
| Neural Network | 0.731 (0.717-0.745) | 0.700 (0.683-0.717) | 0.693 (0.672-0.714) | 0.753 (0.727-0.779) | 0.778 (0.747-0.810) |

The macro AUC was calculated from the averaging the AUC for each class. The AUC for each class was derived using a one versus all approach, which collapses each class into a binary outcome, e.g. having neither suicidal behaviours nor aggressive behaviours vs all other outcomes combined

**S9 Table.** Confusion matrices for the datasets

|  | Predicted Neither (proportion of true set) | Predicted Suicidal Behaviours (proportion of true set) | Predicted Aggressive Behaviours (proportion of true set) | Predicted Both (proportion of true set) |
| --- | --- | --- | --- | --- |
| Training set |  |  |  |  |
| True Neither (N = 3 578) | 1 695 (47.25%) | 1 026 (28.60%) | 676 (18.85%) | 190 (5.30%) |
| True Suicidal Behaviours (N = 617) | 139 (22.52%) | 326 (52.84%) | 65 (10.53%) | 87 (14.10%) |
| True Aggressive Behaviours (N = 348) | 42 (12.97%) | 44 (12.64%) | 170 (48.45%) | 92 (26.44%) |
| True Both (N = 221 ) | 41 (18.55%) | 32 (14.48%) | 43 (19.46%) | 105 (47.51%) |
| Tune set |  |  |  |  |
| True Neither (N = 437 ) | 200 (45.77%) | 118 (27.00%) | 88 (20.14%) | 31 (7.09%) |
| True Suicidal Behaviours  (N = 89) | 24 (26.97%) | 42 (47.19%) | 10 (11.24%) | 13 (14.61%) |
| True Aggressive Behaviours (N = 47 ) | 10 (21.28%) | 8 (17.02%) | 21 (44.68%) | 8 (17.02%) |
| True Both (N = 30) | 8 (26.67%) | 12 (40.00%) | 2 (6.67%) | 8 (26.67%) |
| CATSS test set |  |  |  |  |
| True Neither (N = 449) | 199 (44.32%) | 135 (30.07%) | 86 (19.15%) | 29 (6.46%) |
| True Suicidal Behaviours  (N = 66) | 13 (19.50%) | 31 (46.97%) | 10 (15.15%) | 12 (18.18%) |
| True Aggressive Behaviours (N = 52) | 21 (40.38%) | 6 (11.54%) | 11 (21.15%) | 14 (26.92%) |
| True Both (N = 31) | 4 (12.90%) | 8 (25.81%) | 14 (45.16%) | 5 (16.13%) |
| External validation (NTR) set |  |  |  |  |
| True Neither (N = 2 244) | 990 (44.12%) | 694 (30.93%) | 429 (19.12%) | 131 (5.84%) |
| True Suicidal Behaviours (N = 85) | 26 (30.59%) | 30 (35.29%) | 19 (22.35%) | 10 (11.76%) |
| True Aggressive Behaviours (N = 327) | 53 (16.21%) | 71 (21.71%) | 91 (27.83%) | 112 (34.25%) |
| True Both (N = 46) | 9 (19.57%) | 13 (28.26%) | 7 (15.22%) | 17 (36.96%) |

As prediction models give probabilities of belonging to each class rather than binary predictions, thresholds for determining classification must be chosen. This threshold was calculated based on the classifying a preset proportion of participants in the different classes. In other words, 40% of the participants would be classified as having neither outcome, 30% as having suicidal behaviours, 20% as having aggressive behaviors, and 10% as having both. This proportion was decided over using the true proportion based on it’s classification performance in the training set. From this predetermined proportion the thresholds for each class were selected automatically.

**S10 Table.** Variable importance scores for random forest and gradient boosted machines

|  | | **Random Forest** | | **Gradient Boosted Machines** | |
| --- | --- | --- | --- | --- | --- |
| Variable | Average Scaled Importance | Relative Importance | Scaled Importance | Relative Importance | Scaled Importance |
| Aggression symptoms w2 | 0.980 | 1956.51 | 1.00 | 775.23 | 0.96 |
| Sex | 0.920 | 1642.65 | 0.84 | 810.57 | 1.00 |
| Externalizing w2 | 0.640 | 1296.65 | 0.66 | 500.95 | 0.62 |
| Internalizing symptoms w2 | 0.610 | 1186.24 | 0.61 | 495.73 | 0.61 |
| Social problems w2 | 0.400 | 981.56 | 0.50 | 241.69 | 0.30 |
| General Psychopathology PGS | 0.385 | 922.36 | 0.47 | 239.32 | 0.30 |
| ADHD symptoms w2 | 0.330 | 684.22 | 0.35 | 249.28 | 0.31 |
| Anxiety PGS | 0.255 | 526.64 | 0.27 | 192.11 | 0.24 |
| Population stratification PC 2 | 0.255 | 540.00 | 0.28 | 185.15 | 0.23 |
| Anorexia nervosa PGS | 0.245 | 586.09 | 0.30 | 157.80 | 0.19 |
| Post-traumatic stress disorder PGS | 0.235 | 497.54 | 0.25 | 175.60 | 0.22 |
| Schizophrenia PGS | 0.235 | 467.89 | 0.24 | 183.95 | 0.23 |
| Neuroticism PGS | 0.220 | 550.61 | 0.28 | 127.41 | 0.16 |
| IQ PGS | 0.215 | 462.79 | 0.24 | 157.47 | 0.19 |
| Population stratification PC 1 | 0.215 | 476.72 | 0.24 | 154.43 | 0.19 |
| Brith weight PGS | 0.210 | 467.90 | 0.24 | 147.78 | 0.18 |
| Population stratification PC 3 | 0.205 | 456.92 | 0.23 | 148.19 | 0.18 |
| Population stratification PC 5 | 0.200 | 440.66 | 0.23 | 136.34 | 0.17 |
| Aggression PGS | 0.190 | 333.93 | 0.17 | 170.42 | 0.21 |
| ADHD PGS | 0.180 | 331.37 | 0.17 | 155.72 | 0.19 |
| Bipolar disorder PGS | 0.180 | 428.25 | 0.22 | 111.69 | 0.14 |
| Ever been drunk | 0.180 | 490.32 | 0.25 | 87.42 | 0.11 |
| Educational attainment PGS | 0.180 | 413.96 | 0.21 | 118.88 | 0.15 |
| Well-being PGS | 0.180 | 327.60 | 0.17 | 153.62 | 0.19 |
| Childhood BMI PGS | 0.165 | 382.58 | 0.20 | 107.46 | 0.13 |
| Major depressive disorder PGS | 0.150 | 394.80 | 0.20 | 82.86 | 0.10 |
| Autism spectrum disorder PGS | 0.145 | 403.00 | 0.21 | 61.61 | 0.08 |
| ADHD symptoms w1 | 0.140 | 411.22 | 0.21 | 57.73 | 0.07 |
| Ever used marijuana | 0.130 | 232.10 | 0.12 | 116.91 | 0.14 |
| Birth year | 0.125 | 204.78 | 0.10 | 124.03 | 0.15 |
| Family critizes each other w2 | 0.110 | 246.27 | 0.13 | 71.20 | 0.09 |
| Internalizing symptoms w1 | 0.085 | 203.93 | 0.10 | 58.98 | 0.07 |
| Family quarrels often w2 | 0.085 | 137.93 | 0.07 | 77.67 | 0.10 |
| Population stratification PC 4 | 0.080 | 196.31 | 0.10 | 45.34 | 0.06 |
| Externalizing symptoms w1 | 0.050 | 66.80 | 0.03 | 55.34 | 0.07 |
| Family yells w2 | 0.040 | 132.13 | 0.07 | 4.43 | 0.01 |
| Social problems w1 | 0.040 | 119.52 | 0.06 | 15.59 | 0.02 |

Abbreviations:

w1 = wave 1 (age 9/12)

w2 = wave 2 (age 15/16)

PGS = polygenic score

PC = Principal component

**S11 Table**. Model performance for the test and external validation sets for the sensitivity analysis

|  | **AUC (95% CI) ^1^** | **Sensitivity** | **Specificity** | **Positive Predictive Value** | **Negative Predictive Value** | **Youden’s J statistic** |
| --- | --- | --- | --- | --- | --- | --- |
| **CATSS test set** |  |  |  |  |  |  |
| Macro | 0.677 (0.637-0.719) | 0666 | 0.614 | 0.312 | 0.810 | 0.279 |
| Neither | 0.655 (0.602-0.708) | 0.688 | 0.557 | 0.824 | 0.372 | 0.245 |
| Suicidal behaviours | 0.690 (0.624-0.760) | 0.636 | 0.650 | 0.184 | 0.935 | 0.287 |
| Aggressive behaviours | 0.674 (0.600-0.751) | 0.654 | 0.639 | 0.147 | 0.951 | 0.293 |
| Both | 0.690 (0.615-0.769) | 0.806 | 0.564 | 0.092 | 0.982 | 0.371 |
| **External validation (NTR) set** |  |  |  |  |  |  |
| Macro | 0.682 (0.653-0.710) | 0.646 | 0.620 | 0.312 | 0.799 | 0.266 |
| Neither | 0.711 (0.684-0.738) | 0.690 | 0.638 | 0.903 | 0.296 | 0.327 |
| Suicidal behaviours | 0.551 (0.487-0.615) | 0.224 | 0.875 | 0.055 | 0.972 | 0.099 |
| Aggressive behaviours | 0.748 (0.720-0.777) | 0.621 | 0.753 | 0.257 | 0.935 | 0.374 |
| Both | 0.717 (0.647-0.792) | 0.652 | 0.684 | 0.034 | 0.991 | 0.336 |

This model contained no genetic variables

1 Area under the curve (10,000 bootstrap; 95% CI)

Youden’s J statistic was used to determine the optimal sensitivity and specificity. As models give probabilities of belonging to classes rather than specific class predictions, a threshold must be selected at which a participant is classified into a specific class. Youden’s J is a way to determine this threshold by finding the maximum result of the following equation with the values associated with each threshold:

$$sens +spec-1$$

The positive predictive value was determined based on the following equation:

$$\frac{sⅇns \times prevalence}{\left( sens \times prevalence \right)+(\left( 1-spec \right)\times\left( 1-prevalence \right))}$$

The negative predictive value was determine based on the following equation:

$$\frac{spec \times(1-prevalence)}{\left( (1-sens) \times prevalence \right)+(\left( spec \right)\times\left( 1-prevalence \right))}$$

The macro values were taken from the average values of each class, when collapsing each class down to a binary outcome

**S12 Table.** Parameters tried for random forest in the sensitivity analysis

| **Parameters** | **Description** | **Final** | **Range** |
| --- | --- | --- | --- |
| **Mtries** | Number of variables sampled at each node | 4 | 4 - 8 |
| **Max depth** | Maximum number of edges from the first to last node | 4 | 2-5 |
| **Number of trees** | Number of aggregated trees | 1000 | 200-1,400 |
| **Histogram type** | Method to break up continuous variables for the decision process | Round Robin | Round robin, Quantiles global, Automatic |
| **Sample rate** | Number of participants sampled in each tree | 1 | 0.7-1.00 |

Note: A combination of grid search and random search was used for the parameter search. The parameter search was stopped after all possible combinations were tried or if the mean per class error did not improve by at least 0.0001 for 5 rounds

AUC (1000 bootstrap, 95% CIs]) train set: Macro 0.753 (0.740-0.765) ; Neither 0.709 (0.692-0.726); Suicidal behaviours 0.696 (0.675-0.718); Aggressive behaviours 0.757 (0.731-0.784); Both 0.931 (0.915-0.949)

AUC tune set: Macro 0.688 (0.647-0.730); Neither 0.671 (0.623-0.720); Suicidal behaviours 0.636 (0.579-0.694); Aggressive behaviours 0.750 (0.679-0.827); Both 0.848 (0.823-0.874)

**S13 Table.** Parameters for neural network in the sensitivity analysis

| **Parameters** | **Description** | **Final** | **Range** |
| --- | --- | --- | --- |
| **Hidden layers** | The number of nodes and layers | (20, 20) | (20, 20), (10,10,10), (50, 50), (30,30), (15,15,15), (5,5,5) |
| **Initial drop out ratio** | A form of regularization, i.e. minimizing loss, which randomly removes a ratio of layer outputs | 0.05 | 0 - 0.5 |
| **Rate** | Learning rate, the amount the weights are updated in response to the error rate | 0.01 | 0.01 – 0.02 |
| **Activation** | Input signal function for the node | Rectifier with dropout | Rectifier, Maxout, Rectifier with dropout, maxout with dropout |
| **Epoch** | Number of iterations of the dataset | 62 | 1 - 100 |
| **L1** | Lasso regularization | 2.6^-5^ | 0 - 1^-6^ |
| **L2** | Ridge regularization | 10.1^45^ | 0 - 1^-6^ |

Note: A combination of grid search and random search was used for the parameter search. The parameter search was stopped after all possible combinations were tried or if the mean per class error did not improve by at least 0.0001 for 5 rounds

H2o uses forward propagation

AUC (1000 bootstrap, 95% Cis) train set: Macro 0.702 (0.661-0.745); Neither 0.677 (0.632-0.725); Suicidal behaviours 0.691 (0.636-0.748); Aggressive behaviours 0.751 (0.673-0.835); Both 0.689 (0.586-0.799)

AUC tune set: Macro 0.702 (0.661-0.745); Neither 0.677 (0.632-0.725); Suicidal behaviours 0.691 (0.636-0.748); Aggressive behaviours 0.751 (0.673-0.835); Both 0.689 (0.586-0.799)

**S14 Table.** Parameters for gradient boosted machines in the sensitivity analysis

| **Parameters** | **Description** | **Final** | **Range** |
| --- | --- | --- | --- |
| **Max depth** | Maximum number of edges from the first to last node | 3 | 2 - 12 |
| **Number of trees** | Number of aggregated trees | 1,000 | 250-10,000 |
| **Same rate** | Number of participants sampled in each tree | 1 | 0.20-1.00 |
| **Column sample rate** | Number of variables sampled aggregated with sample rate | 1 | 0.20-1.00 |
| **Column sample rate per tree** | Number of variables sampled at each tree, aggregated with column ample rate | 1 | 0.20-1.00 |
| **Column sample rate change per level** | Change of variable sampling per depth of tree | 1 | 0.9-1.10 |
| **Minimum rows** | Minimum number of participants in a node | 10 | 0-12 |
| **Nbins** | Number of bins for each histogram to build | 20 | 4 - 20 |
| **Learn rate annealing** | Rate to reduce to learn rate after each created tree | 1.0 | 0.5-1 |
| **Histogram type** | Method to break up continuous variables for the decision process | Automatic | Automatic, Quantiles global, Round robin |
| **Learn rate** | Learning rate, the amount the weights are updated in response to the error rate | 0.2 | 0.01-1.00 |

Note: A combination of grid search and random search was used for the parameter search. The parameter search was stopped after all possible combinations were tried or if the mean per class error did not improve by at least 0.0001 for 5 rounds.

AUC train set (1000 bootstrap, 95% Cis): Macro 0.792 (0.781-0.804); Neither 0.734 (0.718-0.750); Suicidal behaviours 0.733 (0.713-0.754); Aggressive behaviours 0.809 (0.786-0.832); Both 0.892 (0.873-0.913)

AUC tune set: Macro 0.693 (0.654-0.735); Neither 0.677 (0.631-0.726); Suicidal behaviours 0.654 (0.597-0.711); Aggressive behaviours 0.758 (0.684-0.837); Both 0.684 (0.589-0.784)

**S15 Table.** Parameters for elastic net in the sensitivity analysis

| **Parameters** | **Description** | **Final** | **Range** |
| --- | --- | --- | --- |
| **Alpha** | L1 and L2 regularization distribution | 0 | 0-0.90 |
| **Lambda** | Regularization strength | 0 | 0-0.01 |
| **Theta** | 1/x for the negative binomial family | 1^-10^ | 1^-10^ -0.1 |

Note: A combination of grid search and random search was used for the parameter search. The parameter search was stopped after all possible combinations were tried or if the mean per class error did not improve by at least 0.0001 for 5 rounds

AUC train set (1000 bootstrap, 95% Cis) : Macro 0.689 (0.674-0.704); Neither 0.675 (0.657-0.693); Suicidal behaviours 0.648 (0.625-0.670); Aggressive behaviours 0.706 (0.676-0.735); Both 0.730 (0.696-0.764)

AUC tune set: Macro 0.693 (0.654-0.735); Neither 0.677 (0.631-0.726); Suicidal behaviours 0.654 (0.597-0.711); Aggressive behaviours 0.758 (0.684-0.837); Both 0.684 (0.589-0.784)

**S16 Table.** Results of Venkatraman test for the difference in ROC curves, bootstrap 2000 times

|  | **P-value** |
| --- | --- |
| **CATSS test set** |  |
| Neither | 0.459 |
| Suicidal behaviours | 0.004 |
| Aggressive behaviours | 0.786 |
| Both | 0.786 |
| **External validation (NTR) set** |  |
| Neither | 0.027 |
| Suicidal behaviours | 0.632 |
| Aggressive behaviours | 0.381 |
| Both | 0.081 |

The macro AUC was not tested as it is calculated by averaging the values of each class, thus cannot be tested using the Venkatraman test.

**S17 Table.** Transparent reporting of a multivariable prediction model for individual prognosis or diagnosis (TRIPOD) Checklist

**References**

1. Anckarsäter H, Lundström S, Kollberg L, Kerekes N, Palm C, Carlström E *et al.* The Child and Adolescent Twin Study in Sweden (CATSS). *Twin Research and Human Genetics* 2012; **14**(6)**:** 495-508.

2. Brikell I, Larsson H, Lu Y, Pettersson E, Chen Q, Kuja-Halkola R *et al.* The contribution of common genetic risk variants for ADHD to a general factor of childhood psychopathology. *Molecular psychiatry* 2018**:** 1.

3. Ligthart L, van Beijsterveldt CEM, Kevenaar ST, de Zeeuw E, van Bergen E, Bruins S *et al.* The Netherlands Twin Register: Longitudinal Research Based on Twin and Twin-Family Designs. *Twin Research and Human Genetics* 2019; **22**(6)**:** 623-636.

4. Consortium GP. A global reference for human genetic variation. *Nature* 2015; **526**(7571)**:** 68.

5. Consortium IH. Integrating common and rare genetic variation in diverse human populations. *Nature* 2010; **467**(7311)**:** 52.

6. Chang CC, Chow CC, Tellier LC, Vattikuti S, Purcell SM, Lee JJ. Second-generation PLINK: rising to the challenge of larger and richer datasets. *Gigascience* 2015; **4**(1)**:** s13742-13015-10047-13748.

7. Price AL, Weale ME, Patterson N, Myers SR, Need AC, Shianna KV *et al.* Long-range LD can confound genome scans in admixed populations. *American journal of human genetics* 2008; **83**(1)**:** 132.

8. Larson T, Anckarsäter H, Gillberg C, Ståhlberg O, Carlström E, Kadesjö B *et al.* The autism-tics, AD/HD and other comorbidities inventory (A-TAC): further validation of a telephone interview for epidemiological research. *BMC psychiatry* 2010; **10**(1)**:** 1.

9. Achenbach TM, Edelbrock CS. Manual for the child behavior checklist and revised child behavior profile. 1983.

10. Fowler PC. Maximum likelihood factor structure of the Family Environment Scale. *Journal of Clinical Psychology* 1981; **37**(1)**:** 160-164.

11. Gerard AB. *Parent-child relationship inventory (PCRI)*. Western psychological services1994.

12. Raine A, Dodge K, Loeber R, Gatzke‐Kopp L, Lynam D, Reynolds C *et al.* The reactive–proactive aggression questionnaire: Differential correlates of reactive and proactive aggression in adolescent boys. *Aggressive Behavior: Official Journal of the International Society for Research on Aggression* 2006; **32**(2)**:** 159-171.

13. Stattin H, Kerr M. Parental monitoring: A reinterpretation. *Child development* 2000; **71**(4)**:** 1072-1085.

14. Goodman R. The Strengths and Difficulties Questionnaire: a research note. *Journal of child psychology and psychiatry* 1997; **38**(5)**:** 581-586.

15. Achenbach TM. *Integrative guide for the 1991 CBCL/4-18, YSR, and TRF profiles*. Department of Psychiatry University of Vermont1994.

16. Demontis D, Walters RK, Martin J, Mattheisen M, Als TD, Agerbo E *et al.* Discovery of the first genome-wide significant risk loci for attention deficit/hyperactivity disorder. *Nature genetics* 2019; **51**(1)**:** 63.

17. Ip HF, van der Laan CM, Krapohl EML, Brikell I, Cristina S-M, Nolte IM *et al.* Genetic Association Study of Childhood Aggression across raters, instruments and age. *bioRxiv* 2021**:** 854927.

18. Purves KL, Coleman JRI, Meier SM, Rayner C, Davis KAS, Cheesman R *et al.* A major role for common genetic variation in anxiety disorders. *Molecular Psychiatry* 2020; **25**(12)**:** 3292-3303.

19. Duncan L, Yilmaz Z, Gaspar H, Walters R, Goldstein J, Anttila V *et al.* Significant Locus and Metabolic Genetic Correlations Revealed in Genome-Wide Association Study of Anorexia Nervosa. *American Journal of Psychiatry* 2017; **174**(9)**:** 850-858.

20. Ferreira MAR, Mathur R, Vonk JM, Szwajda A, Brumpton B, Granell R *et al.* Genetic Architectures of Childhood- and Adult-Onset Asthma Are Partly Distinct. *The American Journal of Human Genetics* 2019; **104**(4)**:** 665-684.

21. Grove J, Ripke S, Als TD, Mattheisen M, Walters RK, Won H *et al.* Identification of common genetic risk variants for autism spectrum disorder. *Nature genetics* 2019; **51**(3)**:** 431-444.

22. Stahl EA, Breen G, Forstner AJ, McQuillin A, Ripke S, Trubetskoy V *et al.* Genome-wide association study identifies 30 loci associated with bipolar disorder. *Nature Genetics* 2019; **51**(5)**:** 793-803.

23. Warrington NM, Beaumont RN, Horikoshi M, Day FR, Helgeland Ø, Laurin C *et al.* Maternal and fetal genetic effects on birth weight and their relevance to cardio-metabolic risk factors. *Nature genetics* 2019; **51**(5)**:** 804-814.

24. Vogelezang S, Bradfield JP, Ahluwalia TS, Curtin JA, Lakka TA, Grarup N *et al.* Novel loci for childhood body mass index and shared heritability with adult cardiometabolic traits. *PLOS Genetics* 2020; **16**(10)**:** e1008718.

25. Lee JJ, Wedow R, Okbay A, Kong E, Maghzian O, Zacher M *et al.* Gene discovery and polygenic prediction from a genome-wide association study of educational attainment in 1.1 million individuals. *Nature Genetics* 2018; **50**(8)**:** 1112-1121.

26. Taal HR, St Pourcain B, Thiering E, Das S, Mook-Kanamori DO, Warrington NM *et al.* Common variants at 12q15 and 12q24 are associated with infant head circumference. *Nature Genetics* 2012; **44**(5)**:** 532-538.

27. Savage JE, Jansen PR, Stringer S, Watanabe K, Bryois J, de Leeuw CA *et al.* Genome-wide association meta-analysis in 269,867 individuals identifies new genetic and functional links to intelligence. *Nature Genetics* 2018; **50**(7)**:** 912-919.

28. Howard DM, Adams MJ, Shirali M, Clarke T-K, Marioni RE, Davies G *et al.* Genome-wide association study of depression phenotypes in UK Biobank identifies variants in excitatory synaptic pathways. *Nature communications* 2018; **9**(1)**:** 1470-1470.

29. Nagel M, Jansen PR, Stringer S, Watanabe K, de Leeuw CA, Bryois J *et al.* Meta-analysis of genome-wide association studies for neuroticism in 449,484 individuals identifies novel genetic loci and pathways. *Nature Genetics* 2018; **50**(7)**:** 920-927.

30. Nievergelt CM, Maihofer AX, Klengel T, Atkinson EG, Chen C-Y, Choi KW *et al.* International meta-analysis of PTSD genome-wide association studies identifies sex-and ancestry-specific genetic risk loci. *Nature communications* 2019; **10**(1)**:** 1-16.

31. Pardiñas AF, Holmans P, Pocklington AJ, Escott-Price V, Ripke S, Carrera N *et al.* Common schizophrenia alleles are enriched in mutation-intolerant genes and in regions under strong background selection. *Nature genetics* 2018; **50**(3)**:** 381-389.

32. Baselmans BML, Jansen R, Ip HF, van Dongen J, Abdellaoui A, van de Weijer MP *et al.* Multivariate genome-wide analyses of the well-being spectrum. *Nature Genetics* 2019; **51**(3)**:** 445-451.

33. Caspi A, Houts RM, Belsky DW, Goldman-Mellor SJ, Harrington H, Israel S *et al.* The p factor: one general psychopathology factor in the structure of psychiatric disorders? *Clinical Psychological Science* 2014; **2**(2)**:** 119-137.
